# P-869. Acute Kidney Injury Related to Trimethoprim-Sulfamethoxazole Treatment of Pneumocystis jirovecii Pneumonia: A Retrospective Cohort Analysis

**DOI:** 10.1093/ofid/ofaf695.1077

**Published:** 2026-01-11

**Authors:** Aditya Mantha, Reed Van Hook, Sarah Rhoads, James M Jurica, Rachel Johnson, Bruce McCollister, James Maloney, Andrés F Henao Martínez

**Affiliations:** University of Colorado School of Medicine, Broomfield, CO; University of Colorado, longmont, Colorado; University of Colorado, longmont, Colorado; University of California San Diego, San Diego, California; Colorado School of Public Health, University of Colorado Anschutz Medical Campus, Aurora, Colorado; University of Colorado, longmont, Colorado; Univ. of Colorado Anschutz Medical campus, aurora, Colorado; University of Colorado Anschutz Medical Campus, Aurora, Colorado

## Abstract

**Background:**

High-dose oral trimethoprim-sulfamethoxazole (TMP/SMX) for 21 days is the standard dose to treat *Pneumocystis jirovecii* pneumonia (PJP). There has been scant data assessing morbidity due to adverse renal events from TMP/SMX after hospital discharge, despite known adverse effects of TMP on renal function. We evaluated renal function outcomes and health care utilization in a cohort of non-bone marrow transplant, non-HIV (NTNH) patients with PJP.

**Figure:**
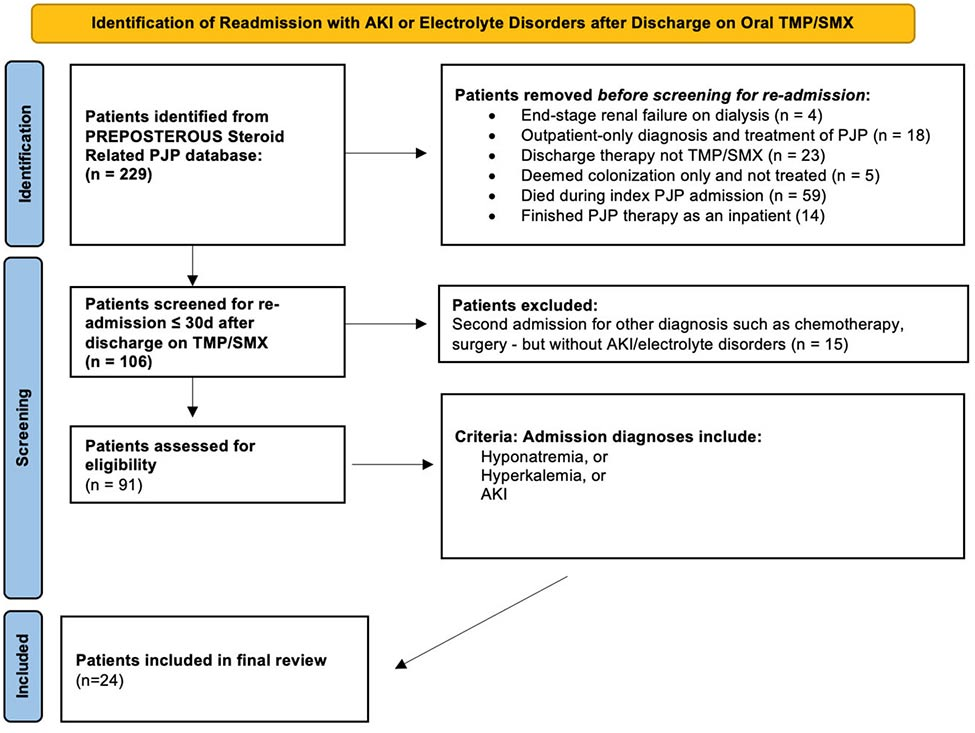

**Figure:**
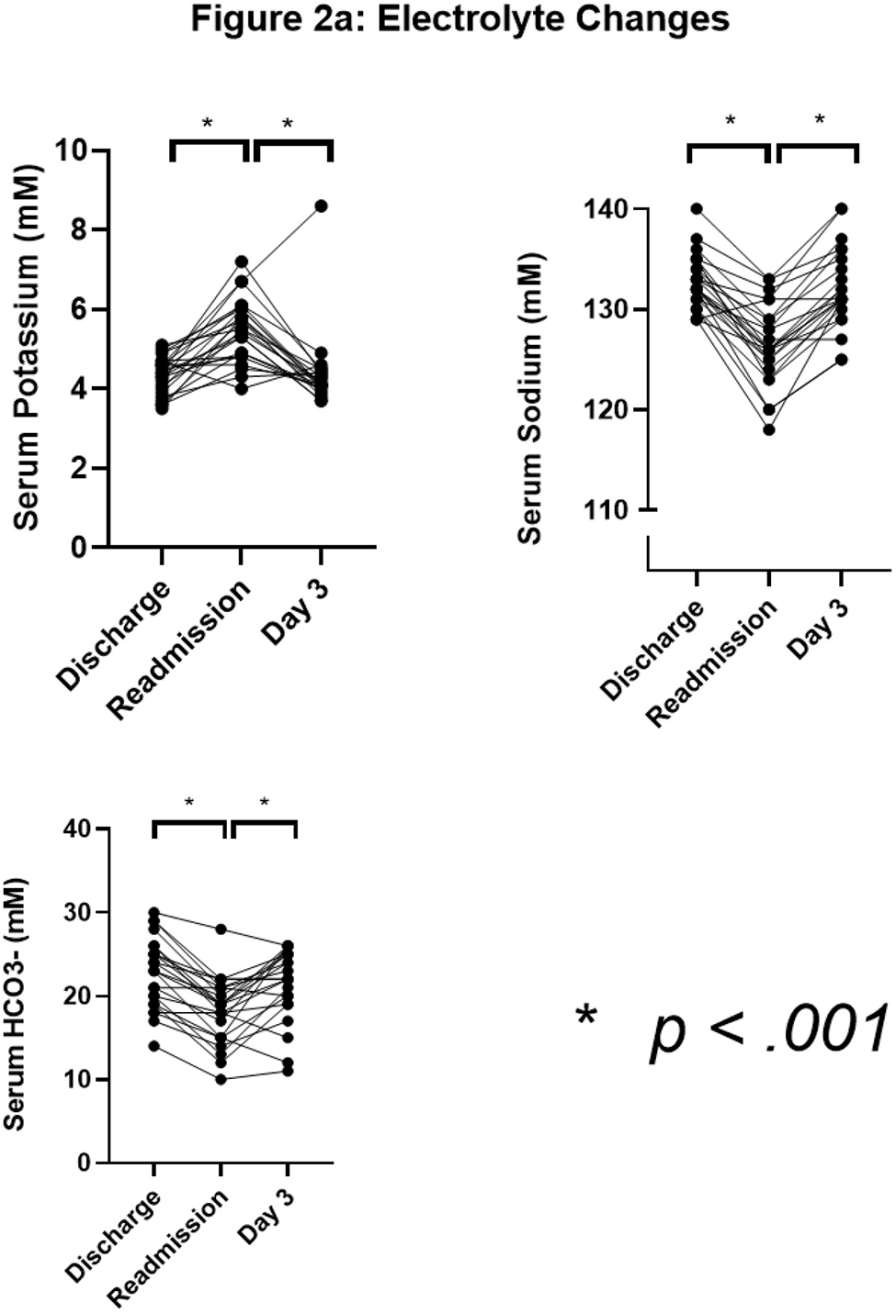

Electrolyte (Na, K, CO3) values for patients on discharge from initial hospitalizations, readmission, and day 3 of readmission

**Methods:**

We conducted a retrospective chart review of NTNH patients diagnosed with PJP at the University of Colorado Hospital (UCH) system over 2016-25 who were admitted for PJP and discharged on high-dose oral TMP/SMX ( >10 mg/kg/day of TMP). We assessed readmission rates due to acute kidney injury (AKI), hyperkalemia, or hyponatremia and compared lab values from discharge, readmission, and 3 days after re-admission by paired t-tests. We used the TriNetX database for national validation with linear mixed models applied to creatinine, sodium, and potassium trajectories by readmission status.

**Figure:**
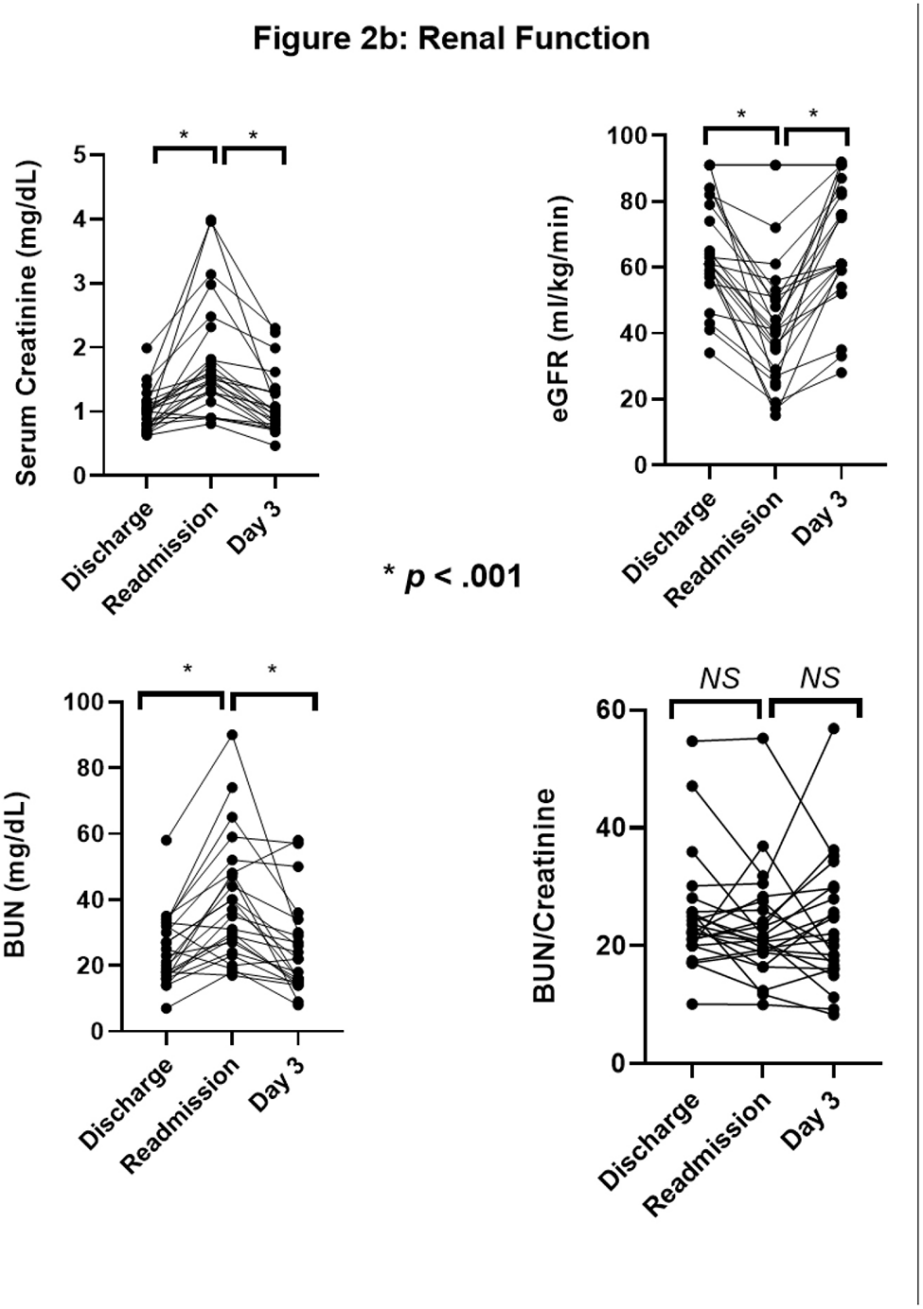
Renal function markers (sCr, eGFR, BUN) for patients on discharge from initial hospitalizations, readmission, and day 3 of readmission

**Figure:**
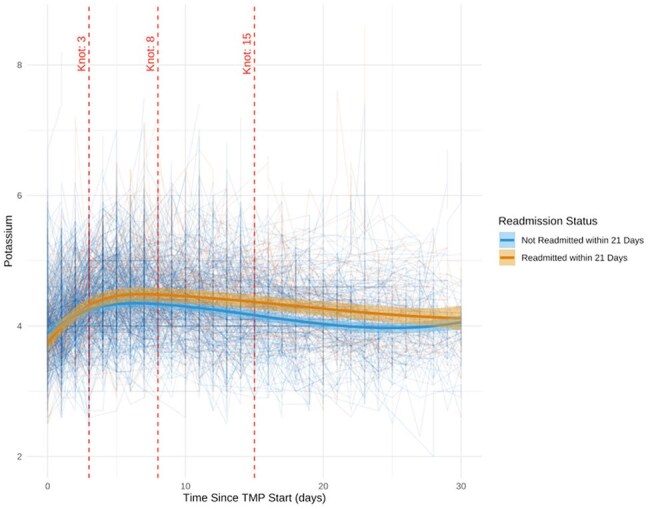
Potassium Trajectory for Patients Who Were Re-admitted and not Re-admitted p <0.001 Change in Potassium for patients who were and were not re-admitted for AKI following TMP-SMX treatment

**Results:**

24 out of 91 UCH patients discharged on TMP/SMX were readmitted for AKI and/or electrolyte disorders during therapy (Figures 1-3). All presented with a KDIGO AKI of stage 1-3 (p< 0.001). Hyperkalemia was observed in 50% of patients (p< 0.001), 42% of whom received shifting therapies. Hyponatremia occurred in 100% (p< 0.001). Symptoms at readmission included weakness (50%), nausea (41.7%), falls (33.3%), and altered mental status (20.8%). One patient died of cardiac arrest attributed to hyperkalemia. We then analyzed 547 NHNT PJP patients in the TriNetX data over 2013-23; 37% were readmitted within 21 days of discharge after PJP diagnosis on TMP/SMX - these patients had similar changes as the UCH cohort with significant impairments in the trajectories of creatinine, potassium, and sodium (p< 0.001) compared to those not readmitted (Figure 4).

**Conclusion:**

High-dose oral consolidation TMP/SMX treatment for PJP results in significant adverse events and healthcare utilization due to a high rate of readmission for AKI - with a number needed to harm of 4. Such outpatient PJP therapy needs closer monitoring than appears to be the current standard-of-care. Clinical trials evaluating efficacy and renal safety of lower-dose TMP/SMX for treating PJP are needed.

**Disclosures:**

Andrés F. Henao Martínez, MD, MPH, F2: Grant/Research Support|Scynexis: Grant/Research Support

